# The Prince and the Hospitals

**Published:** 1900-12-29

**Authors:** 


### The Hospital

A Journal of

The Medical Sciences and Hospital Administration.

Vol. XXIX.—No. 744.] Edited by Sir Henry Burdett, K.C.B., and Solomon C. Smith, M.D., M.R.C.P. [December 29, 1900

### The Prince and the Hospitals.

Lord Lister, in announcing the awards of the Prince of Wales's Hospital Fund for the fourth year on the 18th instant, stated he had never felt so much impressed with the importance and value of the services rendered to the hospitals of London by the Fund as he had been this year. This is unbiassed and important testimony to the usefulness of this Fund and its supreme value to the hospitals. It further illustrates the moral effect the Fund has produced upon numbers of men and women of influence and wealth who had not heretofore taken any active interest in the welfare of the voluntary hospitals. The movement at the outset was based upon the idea of personal service, and such services have been given ungrudgingly by an increasing number of people of all classes, who have become identified with the work of the Prince of Wales's Hospital Fund. Until a few years ago the great bulk of business men actively engaged in the City regarded it as impossible that they could take any personal part in the administration of the hospitals. To-day it is no uncommon thing to find that the partners in the greatest City houses, where time is of paramount value, have associated themselves with the administration of some hospital in London. Lord Lister's words express the very general feeling now current in regard to the Prince's Fund, for all recognise that it has been instrumental for good by arousing the interest and securing the personal services of the most capable and representative of business men. Such results, quite apart from the material aspects of the Fund, would have justified its establishment by showing once more the immense good which the Prince of Wales can accomplish by example in the present day.

But the practical work accomplished by the Prince of Wales's Fund has encouraged and strengthened the hospitals throughout the metropolis. Before the Prince's Fund, no systematic visiting and inspection work was done. Now each year a Visiting Committee of 28 members, including the Presidents of the Royal Colleges of Physicians and Surgeons, of the Royal Society, some of the most representative members of the medical profession, members of the Houses of Lords and Commons, leading merchants, directors of the Bank of England, solicitors and others, organise a thorough inspection of the hospitals of London and devote a good deal of time to the work. The thoroughness of the inspections will be gathered from the fact that the points enquired into cover the systems of management, the hours, work, accommodation, and number of nurses, the state of the buildings generally, the sanitary and general condition of the wards, including ventilation, drainage and protection against fire, the present and past financial condition, including the property, income and expenditure, and the special needs of each institution visited. Experience shows that the visitors are made most heartily welcome, and that every point enquired into is fully appreciated by the authorities who are most anxious to afford the fullest information. The funds entrusted to the Prince of Wales and his Council are therefore not only carefully distributed, but the grants are made out of fulness of knowledge, thanks to the Visiting Committee.

When the Prince's Fund was established, there were not wanting those who publicly stated that it would do harm, that its income could not be maintained, and that the contributions received would gradually fall away to an inconsiderable sum. The fourth report of the Distribution Committee shows that these anticipations have not been fulfilled. During the present year, probably the worst which the home charities have experienced during the last half of the century, the annual subscriptions to the Fund have increased by £1,200, and the revenue at the present time is greater than it has yet been in any previous year. The money distributed this year amongst the hospitals and convalescent institutions amounts to £50,000, which is £8,000 more than was distributed last year, and nearly £20,000 more than the sum at the disposal of the Distribution Committee two years ago. The Prince of Wales's Fund has undoubtedly come to stay. The moral effect upon the hospitals has been wholly beneficial; the visitation of the hospitals has put heart into the managers of every institution inspected, from the smallest to the largest; and the material benefits conferred on the hospitals by the Fund are so great in a year like the present, that without the aid thus received many of the hospitals must have diminished the amount of work done, to the permanent injury of the poorer residents of the metropolis of the Empire. Such results, so secured, cannot fail to give pleasure to the Prince of Wales. They must make everybody grateful to His Royal Highness for having instituted the Fund, which by his personal example, and the cordial co-operation of the Princess of Wales, has become the splendid success which it undoubtedly is to-day. It rests with the people of London to determine to what extent that success shall continue and increase during the twentieth century upon which we are about to enter.

